# Refining the diagnosis of house dust mite-induced allergic rhinitis: optimizing SPT and sIgE cutoff values as predictors of clinically relevant allergy

**DOI:** 10.1038/s41598-026-44756-2

**Published:** 2026-04-10

**Authors:** Lobna A. El-Korashi, Noha M. Hammad, Tarek Gheith, Iman Mohamed Abdel Fattah Ouda, Nessma Hessin Mohamed Gandor, Samar A. Abdelsalam, Ahmed Nagy Hadhoud, Doaa alhussein Abo-alella

**Affiliations:** 1 Department of Medical Microbiology and Immunology, Faculty of Medicine, Zagazig, University, Zagazig, 44519, 44519 Egypt; 2 Al Jouf Regional Laboratory, Shared Services, Ministry of Health, Sakaka, 72311 Saudi Arabia; 3 Department of Clinical Pathology, Faculty of Medicine, Zagazig University, Zagazig, 44519 Egypt; 4 Department of Family medicine, Faculty of Medicine, Zagazig University, Zagazig, 44519 Egypt; 5 Otorhinolaryngology Department, Faculty of Medicine, Zagazig, University, Zagazig, 44519 Egypt

**Keywords:** House dust mites, Der p and Der f, SPT, SIgE, NPT, Immunology, Medical research

## Abstract

Skin prick testing (SPT) and serum-specific IgE (sIgE) measure sensitization rather than clinical allergic rhinitis (AR), making nasal provocation testing (NPT) the reference standard for confirming clinically relevant allergy. A cross-sectional study was conducted on 122 adult AR patients (18–60 years). Participants underwent SPT, sIgE measurement, and NPT for Der p and Der f. Correlations between SPT, sIgE, and NPT results were analyzed, and receiver operating characteristic (ROC) analysis was performed to determine optimal diagnostic cutoffs. Among participants, 106 (86.9%) were SPT-positive for Der p and 80 (65.6%) for Der f. Using the conventional screening threshold (sIgE ≥ 0.35 IU/mL), 86 (70.5%) were positive for Der p and 60 (49.2%) for Der f. NPT positivity was observed in 88 (72.1%) patients for Der p and in 68 (55.7%) patients for Der f. ROC analysis identified higher cutoffs associated with improved specificity: ≥4.2 mm mean wheal diameter (MWD) for SPT and ≥ 1.10 IU/mL for sIgE in Der p, and ≥ 3.75 mm MWD and ≥ 0.50 IU/mL for Der f. While the conventional sIgE threshold (≥ 0.35 IU/mL) remained a strong independent predictor of NPT positivity, the ROC-derived cutoffs demonstrated enhanced diagnostic precision. Our results suggest that although conventional thresholds remain appropriate for initial screening, the ROC-optimized values identified in this study provide greater diagnostic precision and may aid clinical decision-making when NPT is unavailable.

## Introduction

Allergic rhinitis (AR) is a prevalent inflammatory disorder of the nasal mucosa triggered by allergen exposure, affecting millions worldwide and significantly impacting quality of life. House dust mites (HDM), particularly *Dermatophagoides pteronyssinus* (Der p) and *Dermatophagoides farinae* (Der f), are among the most common allergens associated with AR. Accurate diagnosis of HDM-induced AR is essential for effective management, including allergen avoidance strategies and allergen-specific immunotherapy (AIT)^1,2^.

The standard diagnostic approach for AR includes clinical history, skin prick testing (SPT), and serum-specific IgE (sIgE) measurement. While SPT and sIgE are widely used to assess allergen sensitization, they do not always correlate with clinical symptoms, as sensitization does not necessarily indicate clinical allergy. Some patients with positive SPT and sIgE remain asymptomatic, whereas others with AR symptoms have negative results, particularly in cases of local allergic rhinitis (LAR). Therefore, an accurate diagnosis requires confirming clinical reactivity, which sensitization tests cannot establish alone^3^.

Nasal provocation testing (NPT) is the gold standard for diagnosing clinically relevant AR, as it directly assesses nasal mucosal reactivity upon allergen exposure. Unlike SPT and sIgE, which measure systemic sensitization, NPT provides a functional assessment of allergic inflammation in the target organ (nasal mucosa). However, NPT has limitations, including being time-consuming, requiring patient compliance, and lacking widespread availability in routine practice. Despite these drawbacks, it remains the most reliable method for distinguishing true allergy from mere sensitization, making it essential for validating SPT and sIgE cutoff values^4^.

Therefore, this study aims to refine SPT and sIgE cutoff values for HDM-induced AR, using NPT as the reference standard. While previous studies have proposed cutoff values, discrepancies in methodology, population characteristics, and allergen extract standardization have led to inconsistent diagnostic thresholds. By determining optimal SPT and sIgE cutoffs in an adult population and evaluating their agreement with NPT results, this study seeks to improve diagnostic accuracy, reduce misclassification, and enhance clinical decision-making.

## Materials and methods

### Study subjects and design

The sample size was determined based on an expected positive predictive value (PPV) of 85%, considering potential variability in SPT and sIgE results relative to NPT-confirmed HDM-induced AR. A more conservative PPV estimate was used instead of the initially assumed 90% to enhance the study’s validity and generalizability. Using a power of 80% and an alpha level of 0.05, a minimum of 121 patients was required to achieve statistically meaningful comparisons between SPT, sIgE, and NPT outcomes. This adjustment ensures a more robust sample size determination, reducing the risk of overestimating diagnostic accuracy^5^.

This study was conducted in accordance with the principles of the Declaration of Helsinki. Ethical approval was obtained from the institutional review board (ZU-IRB#10848), and all participants provided written informed consent before enrollment. This cross-sectional study recruited one hundred twenty-two adult AR patients (aged 18–60 years). AR was diagnosed according to the Allergic Rhinitis and its Impact on Asthma (ARIA) guidelines^6^.

Clear and stringent exclusion criteria were applied to minimize variability in patient selection. Uncontrolled asthma was defined as frequent exacerbations requiring systemic corticosteroids, persistent daytime symptoms, or reduced lung function (FEV₁ <80% predicted despite treatment). Additional exclusion criteria included chronic rhinosinusitis or nasal polyposis, which could interfere with nasal reactivity assessment; recent antihistamine or corticosteroid use (within four weeks), which could alter SPT and NPT results; and severe systemic diseases or immunodeficiencies, which might affect immune responses. By refining these criteria, this study aims to enhance reproducibility and ensure consistency in patient selection across different clinical settings. Future research could explore a stratified approach based on asthma severity to assess its impact on diagnostic test performance.

Demographic and clinical data, including sex, age, and other allergic conditions, were recorded. SPT, serum sIgE testing, and NPT for HDM were performed on the participants based on availability and feasibility.

### Evaluation of AR severity

The severity of AR was assessed using the visual analog scale (VAS), which measures the severity of nasal and non-nasal symptoms. Patients with AR were asked to evaluate their nasal and non-nasal overall symptoms on a scale ranging from 0 to 10 cm (Mild: 0–3; Moderate: 3.1–7; Severe: 7.1–10)^6^.

### Skin prick test (SPT)

SPT was conducted by applying blood lancet pricks to the volar aspect of the forearm. This involved using normal saline (negative control), 0.1% histamine (positive control), and Der p and Der f. extracts at concentrations of 30000AU/ml for each [Omega Laboratories Limited, Canada]. After 15 min, the wheel size was measured, and the mean of the longest diameter plus the perpendicular diameter was recorded as mean wheal diameter (MWD). A wheel size 3 mm larger than the negative control was considered a positive result^7^.

### Serum sample collection

A 10 mL venous blood sample was collected via clean venipuncture under aseptic conditions. The sample was then centrifuged at 1800 g for 15 min to separate the serum, which was subsequently stored at −20 °C until further measurement of serum-specific IgE (sIgE) levels for inhalant allergens.

### Serum levels of specific IgE

The immunoblotting technique was performed according to the manufacturer’s guidelines using the AllergyScreen^®^ system (MEDIWISS Analytic GmbH, Moers, Germany) to measure serum-specific IgE (sIgE) levels for 14 prevalent aeroallergens, including *Dermatophagoides pteronyssinus*,* Dermatophagoides farinae*,* Penicillium notatum*,* Alternaria*,* Aspergillus niger*,* Aspergillus fumigatus*,* mixed grasses*,* Candida*,* feather*,* birch*,* latex*,* cat and dog epithelium*,* and cockroach*. The serum sIgE levels were analyzed using the Improvio Scanner System (Moers, Germany). A positive result was a sIgE level of ≥ 0.35 IU/mL.

### HDM Nasal provocation tests

NPTs for Der p and Der f were performed on all participants following the EAACI position paper on the standardization of nasal allergen challenges. Allergen extracts were obtained from Omega Laboratories Limited, Canada, at a 30,000 AU/mL concentration. All allergen extracts used for nasal provocation tests were standardized and subjected to quality control according to the manufacturer’s specifications and current EAACI guidelines.

Prior to NPT, all patients discontinued topical and oral decongestants for one day, intranasal corticosteroids for two weeks, and antihistamines for one week. Baseline nasal symptom scores were recorded, and nasal airflow patency was assessed using peak nasal inspiratory flow (PNIF). As a negative control, each participant received two puffs (50 µL per puff) of a standard saline nasal spray in each nostril. Following this, two puffs (each containing 50 µL) of Der p allergen extract solution were administered to each nostril.

The allergen concentration was gradually increased, starting with a 1:10,000 dilution of the initial prick solution. If no adverse reaction occurred, the dilution was progressively increased in 10-fold increments until reaching 1:1 concentration. Each dose was administered at 15-minute intervals, with one puff directed at the inferior meatus and the other at the middle turbinate. Patients were instructed to inhale deeply before administration, hold their breath during application, and exhale forcefully afterward. Visual Analog Scale (VAS) scores were used for subjective assessment, while PNIF measurements objectively evaluated nasal airflow.

A positive NPT was defined as meeting any of the following criteria:

- Significant objective response: PNIF decreased by ≥ 40% from baseline.
- Strong subjective response: VAS symptom score ≥ 55 mm.
- Moderate combined response: PNIF decrease ≥ 20% with VAS symptom score ≥ 23 mm.

To prevent persistent nasal symptoms following the test, patients were monitored for 30 min. Oral antihistamines, nasal saline irrigation, or oral decongestants were provided for symptom relief^8–11^. After two to four weeks, patients underwent repeat NPT using Der f allergen extract, following the same protocol as the Der p NPT.

### Statistical analysis

Data management was performed using the IBM SPSS Statistics, version 23.0 (IBM Corp., Armonk, NY), released in 2015. Quantitative data were expressed as the mean ± standard deviation and median (range), while qualitative data were expressed as absolute frequencies (number) and relative frequencies (percentage). The t-test was used to compare two groups of normally distributed variables. For two groups of non-normally distributed variables, the Mann-Whitney test was employed. Categorical variables were compared using the Chi-square test or Fisher’s exact test. Receiver Operating Characteristic (ROC) curves were constructed, with the true positive rate (sensitivity) plotted on the y-axis and the false positive rate (100-specificity) on the x-axis. Optimal cutoff values were determined using the Youden index. Spearman’s rank correlation was calculated to assess the correlation between various study variables. Kappa coefficient was calculated to evaluate the agreement between various study variables. Univariate and multivariate logistic linear regression models defined the association between the independent and dependent variables: odds ratios (OR) and 95% confidence intervals (95% CI). Hosmer and Lemeshow test was performed to define model fit. Variables with a p-value < 0.05 in univariate analysis were included in the multivariable logistic regression model. Factors such as polysensitization and co-sensitization to Der p and Der f were examined but were not significant and thus excluded from the final model.

## Results

### Demographic and clinical characteristics

A total of 122 adult patients diagnosed with AR were included in this study (Table 1). The mean age of participants was 33.53 ± 9.31 years, with 53 (43.4%) males and 69 (56.6%) females. Among the total cohort, 47 (38.5%) had bronchial asthma, 12 (9.8%) had eczema, 5 (4.1%) had allergic conjunctivitis, and 4 (3.3%) had urticaria. Notably, only 4 (3.3%) participants in our cohort showed no evidence of allergen sensitization by either SPT or sIgE. Based on the VAS severity score, 19 cases (15.6%) were classified as mild, 80 cases (65.6%) as moderate, and 23 cases (18.8%) as severe.

**Table 1:** Baseline characteristics of the studied population.

Characteristics	*N* = 122
Age (years) Mean ± SD	33.53 ± 9.31
Sex Male Female	53 (43.4%) 69 (56.6%)
**Positive Family history of allergy** Positive Negative	68 (55.7%) 54 (44.3%)
**Associated allergic comorbidities** Asthma Eczema Allergic conjunctivitis Urticaria	47 (38.5%) 12 (9.8%) 5 (4.1%) 4 (3.3%)
**Allergen sensitization to Der p and/or Der f** Present Absent	118 (96.7%) 4 (3.3%)
**VAS Severity Score** Median (range) Mild (0–3) Moderate (3.1–7.0.1.0) Severe (7.1–10.0)	5 (2–10) 19 (15.6%) 80 (65.6%) 23 (18.8%)
**Total IgE (IU\mL)** Median (range)	172.67 (24.88–1276)
**SPT Der p (mm)** MWD Median (range) Positive (≥ 3.0)	5.75 (0.00–12.00) 106 (86.9%)
**SPT Der f** MWD Median (range) Positive (≥ 3.0)	3.35 (0.00–12.0.0) 80 (65.6%)
**sIgE Der p (IU\mL)** Median (range) Positive (≥ 0.35)	1.49 (0.00–100.00) 86 (70.5%)
**sIgE Der f (IU\mL)** Median (range) Positive (≥ 0.35)	0.325(0.00–100.00) 60 (49.2%)
**NPT** Positive Der p Positive Der f	88 (72.1%) 68 (55.7%)

### Sensitization and NPT Results

The SPT results showed that 106 patients (86.9%) were positive for Der p (median MWD: 5.75 mm [range: 0.00–12.00]) and 80 patients (65.6%) were positive for Der f (median MWD: 3.35 mm [range: 0.00–12.00]). Median histamine wheal diameter was 7.3 mm (range 3.50–14.00 mm). All negative controls were negative (no dermographism was reported among our cases). Similarly, 86 patients (70.5%) were positive for sIgE to Der p (median level: 1.49 IU/mL [range: 0.00–100.00]), and 60 patients (49.2%) were positive for sIgE to Der f (median level: 0.325 IU/mL [range: 0.00–100.00]). The NPT results were positive in 88 patients (72.1%) for Der p and 68 patients (55.7%) for Der f (Table 1).

### Comparison of SPT and sIgE Results with NPT for Der p senstization

**Fig. 1:**
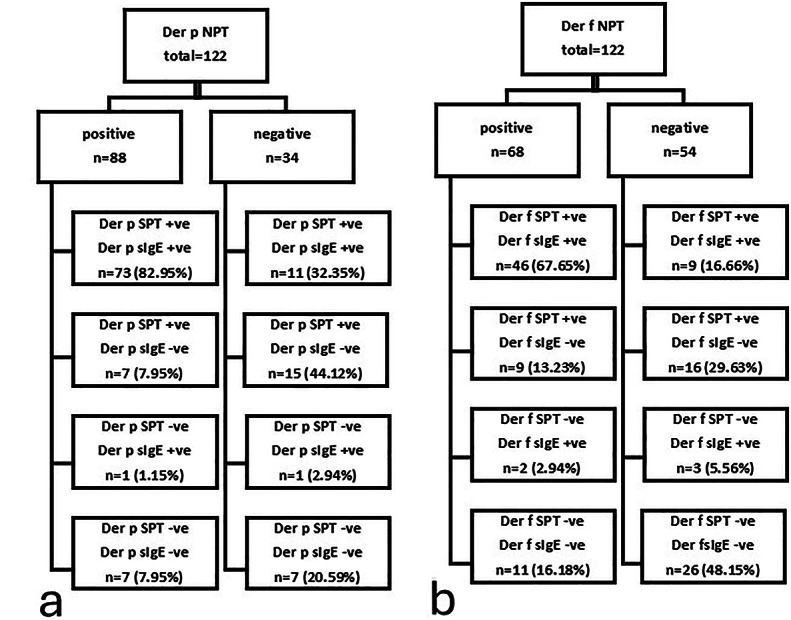
Stratification diagram illustrating nasal provocation test (NPT) results and the distribution of corresponding skin prick test (SPT) and serum specific IgE (sIgE) results among the study population (*N* = 122). Panel (a) represents *Dermatophagoides pteronyssinus* (Der p), and panel (b) represents *Dermatophagoides farinae* (Der f).

Among the 88 patients with positive Der p NPT results, 73 (82.95%) demonstrated positive sensitization by both SPT and sIgE, 7 (7.95%) were positive for SPT only, 1 (1.15%) was positive for sIgE only, and 7 (7.95%) showed no evidence of sensitization via either test. Conversely, among the 34 patients with a negative Der p NPT, 11 (32.35%) had positive sensitization for both SPT and sIgE, 15 (44.12%) were positive for SPT only, 1 (2.94%) was positive for sIgE only, and 7 (20.59%) had no evidence of sensitization (Fig. 1A).

Patients with positive Der p NPT were significantly associated with asthma (61.7% vs. 38.3%, *p* = 0.042), positive Der p SPT results (*p* = 0.034), and elevated Der p sIgE levels (*p* < 0.001) compared to those with negative NPT results. Additionally, they had significantly larger MWDs for Der p SPT (6.50 mm [range: 0.00–12.00] vs. 3.00 mm [range: 0.00–8.00], *p* < 0.001) and higher median Der p sIgE levels (3.08 IU/mL [range: 0.00–100.00 vs. 0.22 IU/mL [range: 0.00–4.40], *p* < 0.001) (Table 2).

**Table 2:** Comparison of clinical and allergen sensitization profiles stratified by Der p NPT results.

Characteristics	NPT Der *p* *N* = 122		Test of Significance	*p*-value
	Positive *n* = 88	Negative *N* = 34		
**Age (years)** Mean ± SD	33.03 ± 8.93	34.79 ± 10.26	t	0.351
**Sex** Male *n* = 53 Female *n* = 69	35 (66%) 53 (76.8%)	18 (34%) 16 (23.2%)	χ ^2^	0.188
**Positive Family history of allergy** Positive *n* = 68 Negative *n* = 54	49 (72.1%) 39 (72.2%)	19 (27.9%) 15 (27.8%)	χ ^2^	0.820
**Associated allergic comorbidities** Asthma *n* = 47 Eczema *n* = 12 Allergic conjunctivitis *n* = 5 Urticaria *n* = 4	29 (61.7%) 9 (75.0%) 4 (80.0%) 2 (50.0%)	18 (38.3%) 3 (25.0%) 1 (20.0%) 2 (50.0%)	χ ^2^ Fisher Fisher Fisher	0.042* 0.990 0.990 0.310
**Allergen sensitization** Senstiztion to Der p and/or Der f *n* = 118 Non- sensitization to any allergen *n* = 4	86 (72.9%) 2 (50.0%)	32 (27.1%) 2 (50.0%)	Fisher	0.310
**VAS Severity Score** Median (range) Mild (0–3) *n* = 19 Moderate (3.1–7.0.1.0) *n* = 80 Severe (7.1–10.0) *n* = 23	5 (2–10) 12 (63.2%) 60 (75.0%) 16 (69.6%)	5 (2–9) 7 (36.8%) 20 (25.0%) 7 (30.4%)	U χ ^2^	0.223 0.559
**Total IgE (IU\mL)** Median (range)	172.7 (24.9–1276.0)	177.0 (26.5–478.0)	U	0.749
**SPT Der p (mm)** MWD Median (range) Positive (≥ 3.00) *n* = 106	6.5.0 (0.00–12.00) 82 (75.5%)	3.00 (0.00–8.00) 27 (24.5%)	U χ ^2^	< 0.001* 0.034*
**SPT Der f (mm)** MWD Median (range) Positive (≥ 3.00) *n* = 80	3.35 (0.00–12.00) 59 (73.8%)	3.25 (0.00–8.00) 21 (26.2%)	U χ ^2^	0.157 0.580
**sIgE Der p (IU\mL)** Median (range) Positive (≥ 0.35) *n* = 86	3.08 (0.00–100.00) 74 (86.0%)	0.22 (0.00–4.40) 12 (34.0%)	U χ ^2^	< 0.001* < 0.001*
**sIgE Der f (IU\mL)** Median (range) Positive (≥ 0.35) *n* = 60	0.31 (0.00–100.00) 41 (68.3%)	0.36 (0.00–22.30) 19 (31.7%)	U χ ^2^	0.559 0.357

*Significant difference.

χ2; Chi-square test, t; Student’ t-test, U; Mann Whitney U test.

### Diagnostic Performance of SPT and sIgE in Der p–Induced AR

**Fig. 2:**
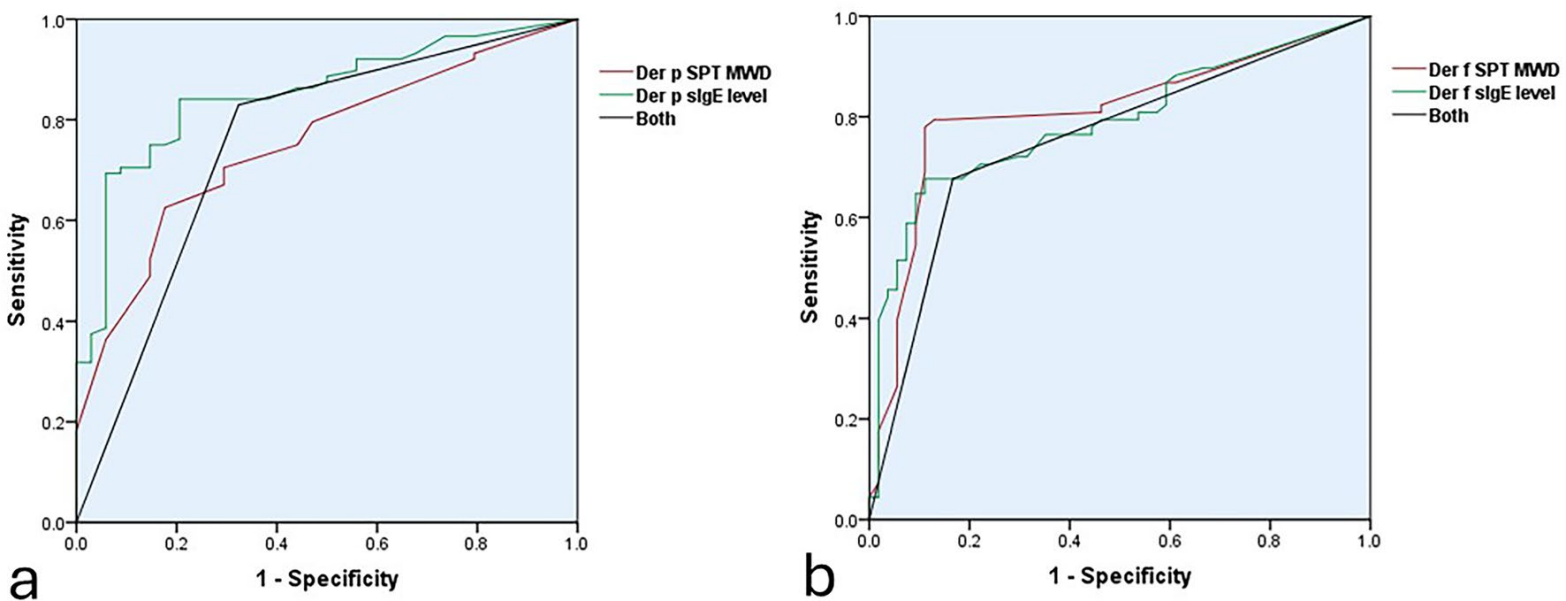
Receiver operating characteristic (ROC) curve analysis of skin prick test (SPT) mean wheal diameter (MWD) and serum specific IgE (sIgE) levels, evaluated individually and in combination, for the diagnosis of (a) *Dermatophagoides pteronyssinus* (Der p) and (b) *Dermatophagoides farinae* (Der f) –induced allergic rhinitis, using the nasal provocation test as the reference standard.

The diagnostic performance of SPT and sIgE for detecting NPT-confirmed Der p allergy is summarized in Table 3. Using conventional thresholds, SPT Der p MWD ≥ 3.00 mm showed high sensitivity (90.9%) but low specificity (23.5%), with a PPV of 75.5%, NPV of 50.0%, and overall accuracy of 72%. Increasing the SPT cutoff to ≥ 4.20 mm improved specificity to 70.6% and PPV to 86.1%, with a sensitivity of 70.5% and accuracy of 70.5%. sIgE Der *p* ≥ 0.35 IU/mL demonstrated a sensitivity of 84.1%, specificity of 64.7%, PPV of 86.0%, NPV of 61.1%, and accuracy of 78.7%. Raising the sIgE cutoff to ≥ 1.10 IU/mL increased specificity to 85.3% and PPV to 93.0%, while sensitivity decreased to 75.0% and accuracy was 77.9%. Combining SPT Der p MWD ≥ 3.00 mm and sIgE ≥ 0.35 IU/mL yielded a sensitivity of 83.0%, specificity of 67.6%, PPV of 86.9%, NPV of 60.5%, and accuracy of 78.7%. The AUC values were 0.753 for SPT, 0.852 for sIgE, and 0.753 for the combined tests (Fig. 2A), indicating good diagnostic discrimination, particularly at optimized cutoffs that enhanced specificity and PPV.

**Table 3:** ROC-derived diagnostic cutoff values of SPT MWD and sIgE levels for NPT-confirmed Der p–induced AR.

Cut off level	Sensitivity	Specificity	PPV	NPV	Accuracy	AUC	95%CI
SPT Der p MWD ≥ 3.00 mm	90.9%	23.5%	75.5%	50.0%	72%	0.753	0.664–0.842
SPT Der p MWD ≥ 4.20 mm	70.5%	70.6%	86.1%	48.0%	70.5%		
sIgE Der *p* ≥ 0.35 IU/Ml	84.1%	64.7%	86.0%	61.1%	78.7%	0.852	0.78–0.923
sIgE Der *p* ≥ 1.10 IUmL	75%	85.3%	93.0%	56.9%	77.9%		
Combined SPT Der p MWD ≥ 3.00 mm and sIgE Der *p* ≥ 0.35 IU/mL	83.0%	67.6%	86.9%	60.5%	78.7%	0.753	0.65–0.856

AUC; Area under the curve, CI; confidence interval, NPV; Negative predictive vaule, PPV; Positive predictive value.

### Comparison of SPT and sIgE Results with NPT for Der f senstization

Among the 68 patients with positive Der f NPT results, 46 (67.65%) demonstrated positive sensitization by both SPT and sIgE, 9 (13.24%) were positive for SPT only, 2 (2.94%) were positive for sIgE only, and 11 (16.18%) had no evidence of sensitization. In contrast, among the 54 patients with negative Der f NPT, 9 (16.66%) were positive for both SPT and sIgE, 16 (29.63%) were positive for SPT only, 3 (5.56%) were positive for sIgE only, and 26 (48.15%) had no evidence of sensitization (Fig. 1B).

Patients with positive Der f NPT had significantly larger median MWDs for Der f SPT (6 mm [range: 0.00–12] vs. 2 mm [range: 0.00–9], *p* = 0.0001) and higher median Der f sIgE levels (1.19 IU/mL [range: 0–100] vs. 0.22 IU/mL [range: 0–32.2], *p* = 0.0001) compared to those with negative NPT results (Table 4).

**Table 4:** Comparison of clinical and allergen sensitization profiles stratified by Der f NPT results.

Characteristics	NPT Der f *N* = 122		Test of Significance	*p*-value
	Positive *n* = 68	Negative *N* = 54		
**Age (years)** Mean ± SD	33.57 ± 9.6	33.46 ± 9.02	t	0.948
**Sex** Male *n* = 53 Female *n* = 69	27 (50.9%) 41 (59.4%)	26 (49.1%) 38 (40.6%)	χ ^2^	0.350
**Positive Family history of allergy** Positive *n* = 68 Negative *n* = 54	36 (52.9%) 32 (59.3%)	32 (47.1%) 22 (40.7%)	χ ^2^	0.56
**Associated allergic comorbidities** Asthma *n* = 47 Eczema *n* = 12 Allergic conjunctivitis *n* = 5 Urticaria *n* = 4	28 (59.6%) 8 (66.7%) 44 (80.0%) 1 (25.0%)	19 (40.4%) 4 (33.3%) 1 (20.0%) 3 (75.0%)	χ ^2^ Fisher Fisher Fisher	0.499 0.420 0.380 0.321
**Allergen sensitization** Senstiztion to Der p and/or Der f *n* = 118 Non- sensitization to any allergen *n* = 4	67 (56.8%) 1 (25.0%)	51 (43.2%) 3 (75.0%)	Fisher	0.321
**VAS Severity Score** Median (range) Mild (0–3) *n* = 19 Moderate (3.1–7.0.1.0) *n* = 80 Severe (7.1–10.0) *n* = 23	5 (2–10) 13 (68.9%) 42 (52.5%) 13 (56.5%)	5 (2–9) 6 (31.6%) 38 (47.5%) 10 (43.5%)	U χ ^2^	0.488 0.453
**Total IgE (IU\mL)** Median (range)	247.54 ± 219.08	178.5 9 ± 105.74	U	0.200
**SPT Der p (mm)** MWD Median (range) Positive (≥ 3.00) *n* = 106	6.00 (0.00–12.00) 61 (57.5%)	5.25 (0.0–11.00) 45 (42.5%)	U χ ^2^	0.288 0.300
**SPT Der f (mm)** MWD Median (range) Positive (≥ 3.00) *n* = 80	6.00 (0.00–12.00) 55 (68.8%)	2.00 (0.00–9.00) 25 (31.2%)	U χ ^2^	< 0.001* < 0.001*
**sIgE Der p (IU\mL)** Median (range) Positive (≥ 0.35) *n* = 86	2.06 (0.00–100.00) 49 (57.0%)	1.16 (0.00–25.49) 37 (43.0%)	U χ ^2^	0.193 0.570
**sIgE Der f (IU\mL)** Median (range) Positive (≥ 0.35) *n* = 60	1.19 (0.00–100.00) 48 (80.0%)	0.22 (0.00–32.20) 12 (20.0%)	U χ ^2^	< 0.001* < 0.001*

*Significant difference.

χ2; Chi-square test, t; Student’ t-test, U; Mann Whitney U test.

Among the 68 patients with positive Der f NPT results, 46 (67.65%) demonstrated positive sensitization by both SPT and sIgE, 9 (13.23%) were positive for SPT only, 2 (2.94%) were positive for sIgE only, and 11 (16.18%) showed no evidence of sensitization. In contrast, among the 54 patients with negative Der f NPT results, 9 (16.66%) demonstrated positive sensitization by both SPT and sIgE, 16 (29.63%) were positive for SPT only, 3 (5.56%) were positive for sIgE only, and 26 (48.15%) showed no evidence of sensitization (Fig. 1B).

Patients with positive Der f NPT results had significantly larger MWDs for Der f SPT (6 mm [range: 0.00–12.00] vs. 2 mm [range: 0.00–90.00], *p* < 0.001) and higher median Der f sIgE levels (1.19 IU/mL [range: 0.00–100.00] vs. 0.22 IU/mL [range: 0.00–32.2.0], *p* < 0.001) compared with those with negative NPT results (Table 4).

### Diagnostic Performance of SPT and sIgE in Der f–Induced AR

The diagnostic performance of SPT and sIgE for detecting NPT-confirmed Der f allergy is summarized in Table 5. Using conventional thresholds, SPT Der f MWD ≥ 3.00 mm showed high sensitivity (80.9%) but moderate specificity (53.7%), with a PPV of 68.8%, NPV of 69.0%, and overall accuracy of 68.8%. Increasing the SPT cutoff to ≥ 3.75 mm improved specificity to 88.9% and PPV to 89.8%, with a sensitivity of 77.9% and accuracy of 82.8%. sIgE Der f ≥ 0.35 IU/mL demonstrated a sensitivity of 70.6%, specificity of 77.8%, PPV of 80.0%, NPV of 67.7%, and accuracy of 73.8%. Raising the sIgE cutoff to ≥ 0.50 IU/mL increased specificity to 88.9% and PPV to 88.5%, while sensitivity decreased to 67.6% and accuracy was 77%. Combining SPT Der f MWD ≥ 3.00 mm and sIgE ≥ 0.35 IU/mL yielded a sensitivity of 67.6%, specificity of 83.3%, PPV of 83.6%, NPV of 67.2%, and accuracy of 74.6%. The AUC values were 0.809 for SPT, 0.789 for sIgE, and 0.755 for the combined tests (Fig. 2B), indicating good diagnostic discrimination, particularly at optimized cutoffs that enhanced specificity and PPV.

**Table 5:** ROC-derived diagnostic cutoff values of SPT MWD and sIgE levels for NPT-confirmed Der f–induced AR.

Cut off level	Sensitivity	Specificity	PPV	NPV	Accuracy	AUC	95%CI
SPT Der f MWD ≥ 3.00 mm	80.9%	53.7%	68.8%	69.0%	68.8%	0.809	0.73–0.89
SPT Der f MWD ≥ 3.75 mm	77.9%	88.9%	89.8%	76.2%	82.8%		
sIgE Der f ≥ 0.35 IU\mL	70.6%	77.8%	80.0%	67.7%	73.8%	0.789	0.707–0.870
sIgE Der f ≥ 0.50 IU/mL	67.6%	88.9%	88.5%	68.6%	77%		
Combined SPT Der f MWD ≥ 3.00 mm and sIgE Der f ≥ 0.35 IU/mL	67.6%	83.3%	83.6%	67.2%	74.6%	0.755	0.667–0.843

AUC; Area under the curve, CI; confidence interval, NPV; Negative predictive vaule, PPV; Positive predictive value.

### Correlation and Agreement between SPT and sIgE for Der p and Der f Sensitization

No significant correlation was found between VAS and SPT MWD, sIgE, or total IgE. However, significant positive correlations were detected between SPT MWDs and sIgE levels for Der p (*r* = 0.486, *p* < 0.001) and Der f (*r* = 0.548, *p* < 0.001) (Table 6). A moderate agreement was observed between SPT and sIgE for both allergens, with a Kappa coefficient of 0.479 (95% CI: 0.31–0.65) for Der p and 0.495 (95% CI: 0.35–0.639) for Der f (Table 7).

**Table 6:** Correlations between measured study variables.

Variables		*r*	*p*-value
Severity (VAS)	SPT Der p MWD	0.126	0.168
Severity (VAS)	sIgE Der p level	0.129	0.157
Severity (VAS)	SPT Der f MWD	0.142	0.120
Severity (VAS)	sIgE Der f level	0.012	0.895
Severity (VAS)	Total IgE level	0.087	0.342
SPT Der p MWD	sIgE Der p level	0.486	< 0.001*
SPT Der f MWD	sIgE Der f level	0.548	< 0.001*

*; Significant difference, r; Spearman Correlation coefficient.

**Table 7:** Kappa Agreement between SPT and sIgE for Der p and Der f Sensitization.

Variables	kappa	95% CI	Agreement
SPT and sIgE Der p	0.479	0.31–0.65	Moderate agreement
SPT and sIgE Der f	0.495	0.35–0.64	Moderate agreement

Cohen’s Kappa: a measure of agreement, ≤ 0: no agreement, 0.01–0.20: slight agreement, 0.21–0.40: fair agreement, 0.41–0.60: moderate agreement, 0.61–0.80: substantial agreement and 0.81–1.00: almost perfect agreement.

### Predictors of Positive Der p NPT

Logistic regression analysis was conducted to identify significant predictors of a positive NPT for Der p (Table 8). Univariate analysis showed that a history of asthma was significantly associated with a lower likelihood of NPT positivity (OR = 0.44, 95% CI: 0.20–0.98, *p* = 0.042). Additionally, patients with SPT positivity for Der p had 3.08 times higher odds of a positive NPT (95% CI: 1.05–9.02, *p* = 0.034). The strongest predictor was sIgE levels ≥ 0.35 IU/mL, which increased the odds of a positive NPT by 9.69 times (95% CI: 3.92–23.98, *p* < 0.001). In the multivariate analysis, sIgE levels ≥ 0.35 IU/mL remained a significant independent predictor of NPT positivity (OR = 10.71, 95% CI: 3.73–30.73, *p* < 0.001), while asthma history (*p* = 0.113) and SPT positivity (*p* = 0.612) were no longer significant. The Hosmer and Lemeshow test showed *p* = 0.122, indicating a good model fit.

**Table 8:** Logistic regression analysis of significant predictors for NPT- confirmed Der P allergy.

Predictor	Univariable Logistic Regression			Multivariable Logistic Regression		
	Exp(B)	95% CI for Exp(B) (Lower - Upper)	*p*-value	Exp(B)	95% CI for Exp(B) (Lower - Upper)	*p*-value
Asthma History	0.44	0.20–0.98	0.042*	0.475	0.19–1.19	0.113
SPT Der p Positive	3.08	1.05–9.02	0.034*	0.709	0.19–2.68	0.612
IgE Der *p* ≥ 0.35 IU/mL	9.69	3.92–23.98	< 0.001*	10.71	3.73–30.73	< 0.001*
Constant				0.09		

**Hosmer and Lemeshow Test**: p = **0.122** (model goodness-of-fit).

*; Significant difference.

### Predictors of Positive Der f NPT

For NPT positivity to Der f, univariate analysis (Table 9) demonstrated that patients with SPT positivity for Der f had 4.91 times higher odds of a positive NPT (95% CI: 2.19–11.00, *p* < 0.001). Similarly, sIgE levels ≥ 0.35 IU/mL were strongly associated with NPT positivity (OR = 8.40, 95% CI: 3.67–19.20, *p* < 0.001). In the multivariate analysis, only sIgE levels ≥ 0.35 IU/mL remained a significant predictor (OR = 6.03, 95% CI: 2.39–15.23, *p* < 0.001), while SPT positivity lost statistical significance (*p* = 0.145). The Hosmer and Lemeshow test showed *p* = 0.271, confirming a good model fit.

**Table 9:** Logistic regression analysis of significant predictors for NPT- confirmed Der f allergy.

Predictor	Univariable Logistic Regression			Multivariable Logistic Regression		
	Exp(B)	95% CI for Exp(B) (Lower - Upper)	*p*-value	Exp(B)	95% CI for Exp(B) (Lower - Upper)	*p*-value
SPT Der p Positive	4.91	2.19–11.00	< 0.001*	2.03	0.78–5.26	0.145
IgE Der *p* ≥ 0.35 IU/mL	8.40	3.67–19.20	< 0.001*	6.03	2.39–15.23	< 0.001*
Constant				−1.05		

**Hosmer and Lemeshow Test**: p = **0.271** (model goodness-of-fit).

*; Significant difference.

## Discussion

SPT and sIgE measurements are widely used to diagnose sensitization to HDM. However, true HDM-induced AR requires sensitization and clinical reactivity, which cannot always be confirmed solely through these tests. NPT remains the gold standard for establishing a causal relationship between allergen exposure and symptoms, but its routine use is limited due to its time-consuming nature and requirement for patient compliance^4^. Therefore, refining the diagnostic accuracy of SPT and sIgE is crucial for correctly identifying patients with clinically relevant HDM-induced AR while minimizing the need for NPT. Our study sought to improve diagnostic precision by determining optimal cutoff values for SPT MWD and sIgE levels in predicting HDM-induced AR, using NPT as the reference standard. While our results demonstrated a strong association between NPT positivity and SPT and sIgE results, discrepancies were noted between these tests. Some patients with positive NPT results exhibited negative SPT or sIgE findings, underscoring the limitations of relying solely on either test for diagnosis. Conversely, false-positive sensitization results highlight the need for appropriate cutoff values to enhance specificity and reduce misclassification^12^.

Our findings offer further clinical validation for SPT and sIgE cutoffs compared to previous studies. Haxel et al. (2016)^13^ reported that an SPT MWD of > 5.00 mm for Der p correlated with NPT positivity in 80% of adult patients. Xiao et al. (2022)^14^ suggested a 5.5 mm cutoff for Der p SPT and a 2.77 IU/mL cutoff for sIgE, with high sensitivity and specificity. However, variations in patient populations and methodologies among studies complicate direct comparisons. Our study refines these thresholds within an adult population, identifying optimal cutoff values of ≥ 4.20 mm for Der p SPT (70.5% sensitivity, 70.6% specificity) and ≥ 1.10 IU/mL for Der p sIgE (75% sensitivity, 85.3% specificity). For Der f, the optimal cutoffs were ≥ 3.75 mm for SPT (77.9% sensitivity, 88.9% specificity) and ≥ 0.50 IU/mL for sIgE (67.6% sensitivity, 88.9% specificity). These values optimize the balance between sensitivity and specificity, improving upon previously reported thresholds and offering refined clinical applicability in an adult population.

Although patients with positive NPT for either Der p or Der f revealed significant associations with positive SPT and elevated sIgE, discrepancies between these tests were observed. Among the 88 patients with a positive Der p NPT, 7 (7.95%) were sensitized based on SPT alone, 1 (1.15%) based on sIgE alone, and 7 (7.95%) had no evidence of sensitization. Similarly, among the 68 patients with a positive Der f NPT, 9 (13.23%) had positive SPT only, 2 (2.94%) had positive sIgE only, and 11 (16.18%) showed no sensitization through either test. These discrepancies may be attributed to false-negative SPT results, which can occur due to reduced skin reactivity, patient-related factors, or variations in allergen extract potency. Conversely, false-negative sIgE results might result from low circulating allergen-specific IgE, poor allergenic extract quality, or sIgE binding to high-affinity receptors on mast cells, making it undetectable in serum. Additionally, LAR cases are, by definition, negative for both SPT and serum sIgE despite clinical allergy confirmed by NPT^12^.

For patients with negative NPT results, 34 with negative Der p NPT showed some degree of sensitization: 11 (32.35%) were positive for both SPT and sIgE, 15 (44.12%) for SPT alone, and 1 (2.94%) for sIgE alone. Similarly, among the 54 patients with negative Der f NPT, 9 (16.66%) had both SPT and sIgE positivity, 16 (29.63%) had positive SPT only, and 3 (5.56%) were positive for sIgE alone. False-positive SPT results could be attributed to urticarial dermographism, atopic dermatitis, or non-specific wheal reactions. In contrast, false-positive sIgE results may arise due to cross-reactivity with homologous allergens or non-specific IgE binding in patients with elevated total IgE levels^15^.

Our study found no significant correlation between the severity of AR and SPT MWD, sIgE, or total IgE levels, which is consistent with previous reports^14,16–18^. However, controversy exists, particularly among children, where some studies have suggested a possible association^5,19^. These discrepancies may be explained by the fact that SPT and sIgE primarily assess sensitization in the skin and blood, whereas AR is fundamentally a respiratory condition. The clinical manifestations of AR depend on multiple factors beyond allergen sensitization, including mucosal inflammation, epithelial barrier integrity, and local immune responses in the nasal mucosa.

Our study demonstrated moderate agreement (k > 0.4) between SPT and sIgE for both Der p and Der f allergens, with a moderate correlation between SPT MWD and sIgE levels (*r* = 0.4 for Der p and *r* = 0.5 for Der f). These correlations were lower than that reported in previous studies. Nam and Lee (2017) found a strong agreement (k > 0.7) and a higher correlation (*r* = 0.7) between SPT and sIgE for both Der p and Der f^20^. Interestingly, their study also noted that correlation strength declined in older patients (> 60 years), suggesting that age-related changes in immune response, skin reactivity, or allergen-specific IgE production may influence diagnostic consistency.

The proposed cutoff values for SPT and sIgE offer practical clinical utility, particularly in settings where NPT is not feasible. Using these optimized cutoff values in primary care and general allergy clinics can improve diagnostic accuracy. Integrating these cutoffs into clinical decision-making algorithms can assist physicians in distinguishing between sensitization and clinically significant allergy, ensuring that AIT is provided to the right patients^12^. Additionally, these findings reinforce the importance of a combined diagnostic approach, as our study demonstrates that SPT and sIgE provide higher specificity and positive predictive value than either test alone. Future studies should explore cost-effectiveness analyses to assess the economic benefits of integrating these cutoff values into routine allergy diagnostics^15^.

Despite its strengths, this study has some limitations. First, the study population was limited to a specific age group and geographical region, which may restrict the generalizability of the findings to other populations with different environmental exposures or genetic backgrounds. Second, LAR was not explicitly analyzed as a subgroup, despite patients with negative SPT and sIgE but positive NPT, highlighting the need for further research on LAR prevalence and its diagnostic markers. Third, the study relied on a single time-point measurement of SPT and sIgE, whereas longitudinal assessments could provide better insights into sensitization dynamics over time. Fourth, the potential influence of co-sensitization to other allergens and cross-reactivity was not extensively explored, which may have contributed to some discrepancies between SPT, sIgE, and clinical symptoms. Lastly, while NPT was used as the reference standard, its availability in routine clinical practice is limited, making it necessary to evaluate whether the proposed SPT and sIgE cutoff values can be reliably used in real-world settings without NPT confirmation.

## Conclusion

This study evaluated the diagnostic performance of SPT and sIgE in predicting NPT-confirmed HDM-induced AR and clarified the clinical positioning of conventional and optimized diagnostic thresholds. Using NPT as the reference standard, we confirmed a strong association between the conventional sIgE threshold (≥ 0.35 IU/mL) and clinically relevant allergy for both *Dermatophagoides pteronyssinus* and *Dermatophagoides farinae*, supporting its role as an accessible and sensitive screening marker. Given its favorable sensitivity and widespread availability, this threshold remains suitable for primary care settings and large-scale risk stratification.

Importantly, ROC analysis identified higher allergen-specific thresholds (e.g., *Der p* sIgE ≥ 1.1 IU/mL and SPT mean wheal diameter ≥ 4.2 mm) that significantly improved diagnostic specificity and positive predictive value. These optimized cutoffs provide incremental diagnostic precision and may be particularly valuable in clinical scenarios requiring greater certainty, such as prior to initiating AIT or in diagnostically challenging cases where minimizing false-positive results is essential.

Together, our findings support a stratified diagnostic approach in which conventional thresholds serve as initial screening tools, while ROC-optimized thresholds enhance confirmatory decision-making when NPT is unavailable or impractical. Future studies should validate these thresholds in broader populations and further refine clinical algorithms integrating sensitization testing with patient-specific risk factors.

## Data Availability

The datasets used and/or analysed during the current study available from the corresponding author on reasonable request.

## Abbreviations

SPT: skin prick tests
sIgE: specific IgE
AR: allergic rhinitis
NPT: nasal provocation test
HDM: house dust mite
Der p: Dermatophagoides pteronyssinus
Der f: Dermatophagoides farina
MWD: mean wheal diameter
VAS: visual analog scale
PNIF: peak nasal inspiratory flows
ROC: Receiver Operating Characteristic
AIT: allergen-specific immunotherapy, LAR, local allergic rhinitis
